# Dataset of Oddball Paradigm experiment in the Auditory Cortex and the effect of acetylcholine

**DOI:** 10.1038/s41597-025-06484-6

**Published:** 2026-01-10

**Authors:** Pablo Vázquez-Borsetti, Ana B. Lao-Rodríguez, Manuel S. Malmierca, David Pérez-González

**Affiliations:** 1 Institute of Cell Biology and Neurosciences (IBCN) – National Scientific and Technical Research Council (CONICET), Buenos Aires, Argentina; 2 Cognitive and Auditory Neuroscience Laboratory (Lab 1), Institute of Neuroscience of Castilla y León (INCYL), University of Salamanca, Salamanca, Spain; 3 Institute for Biomedical Research of Salamanca (IBSAL), Salamanca, Spain; 4 Department of Cell Biology and Pathology, Faculty of Medicine, University of Salamanca, Salamanca, Spain; 5 Department of Basic Psychology, Psychobiology and Methodology of Behavioral Sciences, Faculty of Psychology, University of Salamanca, Salamanca, Spain

**Keywords:** Perception, Neurophysiology, Cortex

## Abstract

This work presents three open datasets featuring various levels of processing, containing neural recordings from the auditory cortex of rats. These recordings were obtained during experiments using the auditory oddball paradigm before, during and after the local microiontophoretic application of acetylcholine. The primary objective of these datasets is to investigate how the brain processes predictable versus unexpected auditory stimuli, and the role of cholinergic inputs during such processing. The data include multi-unit recordings of neuronal activity during the presentation of standard and deviant tones, classified by stimulus type and cortical sub-region. These resources enable quantitative investigations of deviance detection, stimulus-specific adaptation, cholinergic modulation and predictive-coding mechanisms at multiple temporal scales.

## Background & Summary

The auditory cortex is involved in the perception, processing, and interpretation of acoustic information, functioning as a central node for auditory perception, learning, and memory^1–3^. This cortical region dynamically integrates incoming sensory stimuli with contextual information, resulting in perceptual experiences that are finely tuned by various neuromodulatory systems, most notably acetylcholine (ACh)^4,5^. Acetylcholine significantly impacts cortical plasticity, neuronal excitability, attentional processes, and overall sensory processing efficiency^6,7^. ACh inputs to the cortex originate from cholinergic neurons in the basal forebrain^8–10^. Experimental work shows that cholinergic modulation enhances the signal-to-noise ratio in cortical circuits, allowing for improved detection of relevant auditory stimuli^7^, and plays a key role in the attentional modulation of sensory processing^11^.

The predictive coding theory provides a compelling framework for understanding how the auditory system manages sensory information by continuously generating and updating predictions based on previous sensory experiences. This process serves to reduce prediction errors and enhance perceptual accuracy^12,13^. According to these models, the brain encodes expectations about incoming auditory stimuli and compares them with actual sensory input, using mismatches, or prediction errors, as key signals for driving perceptual learning and adaptive neural plasticity^14,15^. Within the predictive coding framework, neuromodulatory systems, particularly cholinergic modulation, are posited to regulate the precision of prediction errors, and thus influencing the weighting and significance assigned to these signals during perceptual inference^16,17^.

Empirical studies across cortical and subcortical levels support these ideas. Adaptation to repetitive stimuli, particularly stimulus-specific adaptation (SSA), has been identified in the auditory cortex^18,19^ and subcortical structures such as the inferior colliculus^20–22^. SSA reflects reduced responses to expected stimuli and preserved responses to rare ones, consistent with the concept of prediction error. The modulation of these responses appears to occur over multiple timescales^19^ and their spatial distribution within the inferior colliculus is both topographically organized and dependent on stimulus frequency and intensity^20,23^. These findings support the view that deviance detection and predictive coding are distributed processes along the auditory hierarchy, as highlighted in comprehensive reviews of the field^24–26^. Experimental studies have consistently demonstrated that acetylcholine plays a pivotal role in modulating neuronal excitability and synaptic plasticity, directly affecting auditory information processing and cortical encoding strategies^5,27^. Elevated cholinergic activity is typically associated with heightened states of attention, improved sensory discrimination, and enhanced adaptability to changing auditory environments, facilitating more accurate prediction formation and error correction^28,29^. Conversely, reduced ACh levels are associated with diminished attentional focus and stabilized cortical representations, effects that are consistent with theoretical predictions about neuromodulatory regulation of uncertainty and sensory precision within predictive coding frameworks^30^.

Investigating the precise interactions between acetylcholine and predictive coding in auditory cortical processing can further shed light in the neural mechanisms underlying sensory perception, adaptive learning, and cognitive disorders involving dysfunctional auditory processing, such as schizophrenia, tinnitus, and auditory processing disorder^31–34^. Hence, a comprehensive characterization of cholinergic modulation within predictive coding circuits of the auditory cortex is a critical step toward uncovering fundamental principles of sensory neuroscience and developing targeted therapeutic interventions for auditory-related disorders.

The contributions of this data to knowledge have already been presented in previous works^35^. In that study, it was demonstrated that the microiontophoretic application of acetylcholine in the rat auditory cortex modulates neuronal mismatch responses by selectively influencing prediction error signaling without affecting repetition suppression. This modulation, predominantly observed in the infragranular layers, suggests that acetylcholine enhances the precision of prediction errors, thereby gating their access to higher cognitive processes. The dataset presented here was originally collected for that study and provides a foundation for further exploration into cholinergic modulation of auditory processing. Ongoing analyses have revealed novel aspects of this process, particularly concerning the effect of the infrequent tone on the restoration of the standard tone (unpublished results). These studies have advanced our understanding of this topic based on the data presented here. However, we believe that additional insights remain to be uncovered, and we hope that colleagues in the field will contribute to further explore and expand upon them.

## Methods

### Surgical procedures

The experimental procedures are detailed in the original publication that included the data^35^. The experimental protocols were approved conforming to the University of Salamanca Animal Care Committee standards and the European Union (Directive 2010/63/EU) for the use of animals in neuroscience research. The study was conducted on 37 adult female Long-Evans rats (180–250 g). Female rats were used based on prior research, without a focus on gender differences. Anesthesia was induced and maintained with urethane (1.5 g/kg, intraperitoneal), with additional doses as needed (0.5 g/kg, intraperitoneal when pedal reflex was observed). Dexamethasone (0.25 mg/kg) and atropine sulfate (0.1 mg/kg) were administered at the start of surgery to reduce brain edema and bronchial secretions. Pain and discomfort were minimized by administering lidocaine around the pinna tissue to achieve a higher level of anaesthesia at the incision sites. The animals were maintained under a deep level of anaesthesia (absence of pedal withdrawal and corneal reflexes, slow and regular respiration, and lack of response to noxious stimuli) throughout the surgery until the endpoint (decapitation).

Once the rat reached the appropriate surgical anesthesia level, the trachea was cannulated for ventilation, and the cisterna magna was drained to prevent brain hernia and edema. Body temperature was maintained at 37–38 °C using a homeothermic blanket, and hydration was ensured with periodic subcutaneous saline injections. The auditory cortex surgery followed procedures described in^22,36^.

### Electrophysiological recording and microiontophoresis

**Fig. 1:**
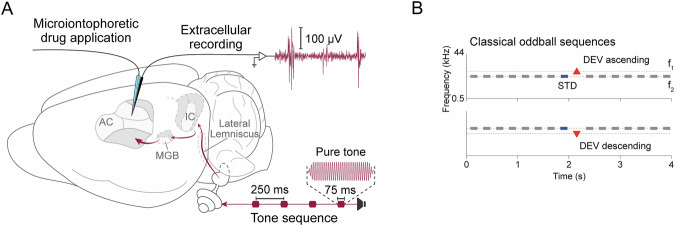
Experimental design. (**A**) Schematic representation of an experimental setup for extracellular recording of auditory-evoked responses from neurons in the rat auditory cortex (AC) during microiontophoretic application of a drug. The recording electrode is attached to the barrels containing the drug; when a current is applied to the barrels, the drug is released next to the recording electrode. Arrows show a simplified ascending auditory pathway from the cochlea to the AC (IC, inferior colliculus; MGB, medial geniculate body of the thalamus). Stimuli were sequences of 75 ms pure tones played by a speaker coupled to one of the ears. (**B**) A classical oddball sequence (top panel) consists of a number of repetitions of a standard tone (STD), with a deviant tone (DEV) at a different sound frequency occurring with low probability. To account for the different responses to both sound frequencies, another oddball sequence was played with the STD and DEV roles inverted (bottom panel).

Multiunit neuronal activity was recorded using a tungsten electrode (1–3 MΩ) attached to a five-barrel borosilicate glass pipette for drug delivery near the recording neuron (Fig. 1A). One of the barrels was filled with saline solution for current compensation (165 mM NaCl) whereas the other barrels were filled with 1 M acetylcholine chloride (Sigma, catalog no. A6625) as a concentration previously used in similar electrophysiological studies^37–39^. Drugs were retained by applying a −15 nA current, and were ejected when required, typically using 30–40 nA currents for 8–10 minutes, until an effect was observed, using a microiontophoresis apparatus (Neurophore BH-2 System, Harvard Apparatus). This technique allows for the local application of the drug (acetylcholine) next to the recording site, thereby affecting only the close vicinity of the recorded neurons.

The electrode assembly was positioned perpendicular to the surface of the auditory cortex and advanced until strong spiking activity, synchronized with search stimuli (white noise), was detected. Signals were digitized at 12 kHz, amplified 251x, and band-pass filtered between 0.5 and 4.5 kHz, using a RZ6 Multi I/O Processor, a RA16PA Medusa Preamplifier, and a ZC16 headstage (Tucker-Davis Technologies, TDT). The recordings were filtered to isolate individual spikes. At the end of the experiment, the data was extracted and processed.

### Experimental design and stimulation paradigms

Sound stimuli were generated using the TDT’s RZ6 Multi I/O Processor and custom software (OpenEx Suite and MATLAB) and presented monaurally in a closed-field condition to the ear opposite the left auditory cortex. The speaker was calibrated to maintain a flat spectrum up to ~75 dB SPL between 0.5 and 44 kHz.

Search stimuli, which included white noise and pure tones, was played while lowering the electrode into the auditory cortex using a controlled manipulator, while monitoring the recorded signal. Upon identifying a suitable neuron, its frequency response area (FRA) was determined using a randomized paradigm that presented tones between 0.5–44 kHz at varying intensities.

During the experiment, oddball sound sequences were played (Fig. 1B). These oddball sequences consisted of frequently repeating standard tones, pseudo-randomly interleaved with rare deviant tones at a different sound frequency (Fig. 1B, top). For each selected pair of stimuli, two oddball sequences (named *oddball* in the data set) with fixed parameters were presented: 400 trials per sequence, 75 ms stimulus duration, 0.5-octave frequency separation, 10% deviant probability, 250 ms onset-to-onset interval, and a minimum of three standard tones before a deviant. In one of the oddball sequences, the tone at the low frequency (f_1_) served as the standard and the high frequency (f_2_) as the deviant; in the other sequence, these roles were reversed. This way, after playing these sequences, we obtained the responses to two tones (f_1_ and f_2_), in two different roles each: deviant and standard.

For each unit, we repeated the same pair of oddball sequences 3 times: a baseline or control condition, before the application of the drug (CTL); a treatment or drug condition, during the application microiontophoretic of acetylcholine (ACh); and a recovery condition, after the effects of the drug have washed out (REC). The REC condition is missing for some of the units, because some units were lost before completing the procedure.

## Data Record

The three datasets, representing neuronal spike data processed at different stages, are publicly available on Figshare^40^. The code and processed datasets can be accessed via the project’s GitHub repository^41^. The raw electrophysiological recordings are hosted on GIN (G-Node) and include a detailed analysis of the spike waveform characteristics^42^.

These datasets contain information on the effects of sound stimulation on the auditory cortex of rats, including the impact of random tone variation, the restoration of the original tone, and the role of cholinergic receptors in these processes.

The dataset comprises recordings from 37 rats, encompassing 114 neurons and a total of 628 recordings (two sequences per neuron). This includes ACh effect recordings, recovery recordings, and baseline data, with removed/excluded recordings.

**Fig. 2:**
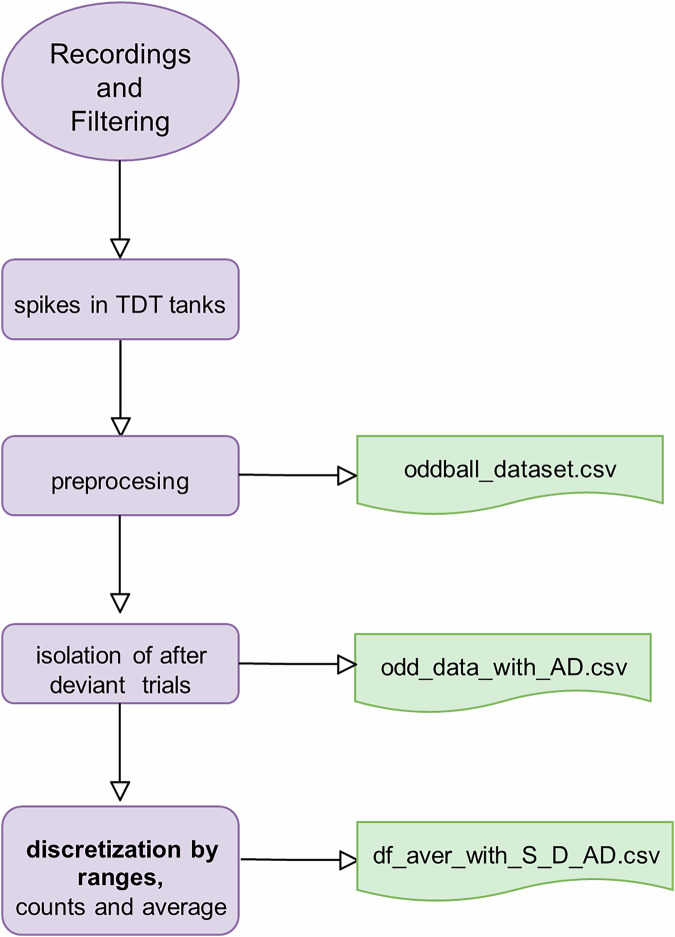
Data Processing Flowchart, indicating the data files obtained at each step.

The datasets are released in CSV format with the following names: *oddball_dataset.csv*, *odd_data_with_AD.csv*, and *df_aver_with_S_D_AD.csv*, each representing different levels of processing (Fig. 2).

The first dataset, *oddball_dataset.csv*, is provided in long format (453809 rows and 18 columns), where each row corresponds to a neuronal spike. The ‘spikes’ column specifies the timing of recorded action potentials relative to the start of the acoustic stimulus in each trial. The second column (‘trial’) indicates the trial number in the experiment. Trials may include multiple spikes, one spike, or none. In trials without spikes, the ‘spikes’ column is left blank in the CSV file. These empty cells are interpreted as NaN during data analysis to indicate the absence of spikes.

The other columns include:

- ‘freq’: Indicates the frequency of the stimulus, in Hz.
- ‘condition’: Encoded marker indicating whether the stimulus is standard (1) or deviant (2).
- ‘animal’: Code indicating the animal (year + animal number). This corresponds to the *tank* name in the raw data, according to TDT’s data nomenclature.
- ‘oddball’: Name of the recorded sequence. The first two digits are a unique identifier for the recording site (tract and neuron). The text string indicates the type of sequence (oddball in this dataset). The next two digits refer to the particular couple of tone frequencies used in the sequence. As mentioned earlier, each neuron is exposed to two tones, one standard and one deviant. The tones (and thus these two digits) are then swapped, so there should be two ‘oddball’ sequences per neuron. The final text string indicates whether this sequence was recorded before (C) or after (F) the application of acetylcholine.
- ‘tract’: Indicates the electrode descent, there can be more than one neuron per tract, and several tracts per animal.
- ‘neuron’: Indicates the recorded neuron in a tract.
- ‘merged_column’: Concatenation of ‘animal’, and ‘oddball’.
- ‘unit’: Concatenation of ‘tract’ and ‘neuron’.
- ‘animal_unit’: Concatenation of ‘animal’ and ‘unit’.

This first dataset also includes spatial information in some recordings, specifically in the column ‘division’, which indicates the field within the auditory cortex. Additionally, three columns provide positional information relative to Bregma:

- ‘depth (Z)’: Vertical position relative to brain surface, in microns.
- ‘rostrocaudal (X)’: Rostro-caudal position relative to bregma, in microns.
- ‘mediolateral (Y)’: Mediolateral position relative to bregma, in microns.

Other parameters included relate to the auditory characteristics of the neuron, such as:

- ‘BF (kHz)’: Best Frequency, the tone frequency that elicits the strongest response from the neuron, in kHz.
- ‘MT (dB)’: Minimum Threshold, the sound intensity at which the neuron starts responding, in dB.

The experiment involving the infusion of acetylcholine (ACh) is differentiated by the column ‘block_type’. In this column, the values *control_block_1* and *control_block_2* correspond to the control conditions, *effect_block_1* and *effect_block_2* represent the recordings during ACh application, and *rec_block_1* and *rec_block_2* indicate the recordings after ACh was cleared, allowing the observation of the recovery of normal neuronal activity.

The second dataset, *odd_data_with_AD.csv*, was derived from the first by assigning a categorical label to the stimulus presented on each trial. It maintains the long-format structure and all original columns, but adds an two extra columns: ‘cat’, indicating the stimulus type (standard, deviant, or after-deviant) and ‘cat2’, where only the standard stimulus previous to a deviant is labelled. As noted earlier, each oddball sequence contains two types of stimuli which differ in their sound frequency: the tone occurring with higher probability is labeled the standard, whereas the lower-probability tone is the deviant. An after-deviant is a particular case of the standard stimulus, corresponding to a standard tone that immediately follows a deviant. This transition is highlighted because many neurons show distinct responses to the shift from deviant to standard.

The third dataset, *df_aver_with_S_D_AD.csv*, was created based on the second dataset. The average neural activity for each oddball sequence was extracted by dividing the trial window into 10 time ranges of 25 ms each, with the column ‘s’ indicating these temporal clusters. E.g., the value 25 refers to the range 0–25 milliseconds relative to stimulus onset.

Additional columns were also included: ‘S_counts’, which represents the total spike count for each time range;’trials’, which indicates the number of trials (for that particular stimulus category) and is used to calculate counts over time and generate a new column, ‘FR(s)’, the average firing rate per second for each time range; ‘FR_agg’, which is the sum of spikes per trial in all time ranges of that stimulus category; ‘mean_standard_FR(s)’, the mean FR(s) for the neuron with standard stimulation before ACh administration; and ‘FR_norm’, which corresponds to the normalized firing rate, adjusted by the average firing rate of the control when stimulated with the standard tone (‘FR(s)’/‘mean FR’).

## Technical Validation

Auditory brainstem responses (ABRs) recorded via subcutaneous electrodes were used to verify normal hearing function prior to surgery. The auditory stimuli consisted of 100 µs clicks at varying sound pressure levels, delivered to the right ear.

The auditory cortex was located using stereotactic coordinates, and a craniotomy was performed. After removing the dura, the exposed cortex was stabilized with agar, and a magnified photograph was taken to map the cortical area. This image was overlaid with a micrometric grid to guide electrode placement during recordings providing spatial information.

The initial steps of preprocessing were carried out during the experiment when the data was acquired. This process included noise filtering and setting parameters for spike discrimination. The data, which was collected using TDT software, was stored in specialized directories called “tanks.” Within these tanks, the data was organized into “blocks,” which were specialized folders that structured the information for further analysis. Each block contained specific details of the electrophysiological experiment. The raw data originally weighed 29.4 GB. To make the data more manageable, the identified neural spikes were extracted with a timestamp. Subsequently, the timing of each recorded spike and its corresponding acoustic stimulation was adjusted. The data was organized into trials, with each trial starting at time 0 at the onset of each acoustic stimulus. Spike times were then reported relative to this reference point.

To ensure the reliability of the data on the effects of acetylcholine (ACh), the experimental design includes controls prior to ACh administration, recordings of the effects during ACh administration, and, in most cases, data capturing the recovery of normal neuronal activity after ACh clearance. This structure enables the verification of the reversible nature of the observed effects.

The preprocessing code also includes automatized recovery, discrimination and inclusion of data of multiple experiments (animals) and experimental units (unitary neurons). The code was developed following the recommendations of for Tucker-Davis Technologies (TDT) Python APIs (https://pypi.org/project/tdt). This code is also available in the preprocessing folder. Some records that showed highly unusual firing patterns were removed.

**Fig. 3:**
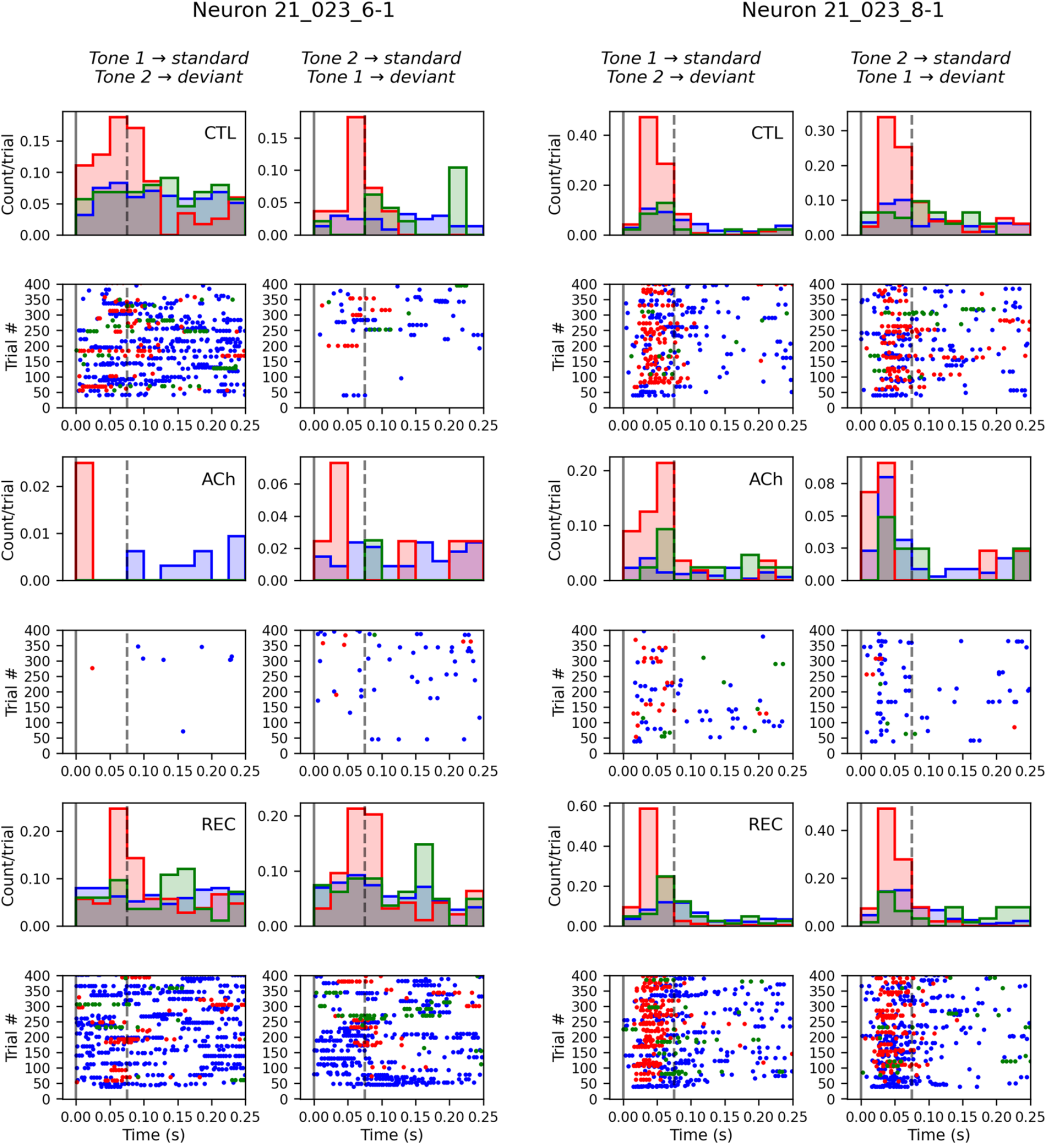
Two representative neurons (‘21_023_6-1’ and ‘21_023_8-1’) illustrate the effect of sound stimulation in the auditory cortex. Blue represents the effect of stimulation with the standard tone, red shows the effect of stimulation with the deviant tone, and green indicates the response to the standard tone immediately following a deviant tone (after deviant). There are two columns per animal, one for each sequence of the oddball stimulation protocol, in which the tone probabilities are swapped. The top two rows show the PSTHs (peristimulus time histogram) and dot rasters of the responses during the control condition (CTL). The following rows depict the effect of acetylcholine (ACh) infusion during the experiment and how the original activity recovers (REC) once the neurotransmitter is washed out. Stimuli start at 0 s and end at 0.075 s, indicated by vertical lines in the plots.

Using this dataset, the main effects of ACh application on neuronal responses in the rat AC during an auditory oddball paradigm can be observed, such as an increase in firing rate (FR) caused by stimulation with the deviant tone (Fig. 3).

**Fig. 4:**
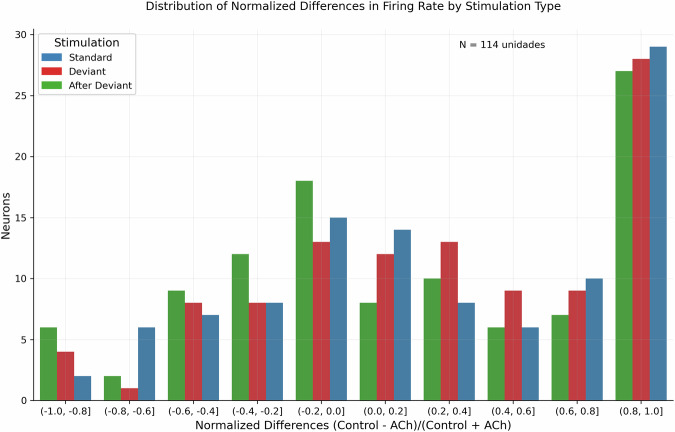
Distribution of normalized differences in firing rate (FR) due to acetylcholine application. A value of 1 on the x-axis indicates a substantial or even complete reduction in FR caused by the microintophoretic release of ACh.

Also, a reduction in FR due to ACh application can be observed. This phenomenon is likewise evident in Fig. 4. To perform an initial descriptive analysis of this phenomenon, the distribution of normalized differences in FR due to acetylcholine can be examined. A value of 1 on the axis indicates a complete reduction in FR during the infusion. The graph suggests the presence of two neuronal populations: one experiencing a substantial decrease in firing rate and another showing a broader effect, with a mean around zero.

**Fig. 5:**
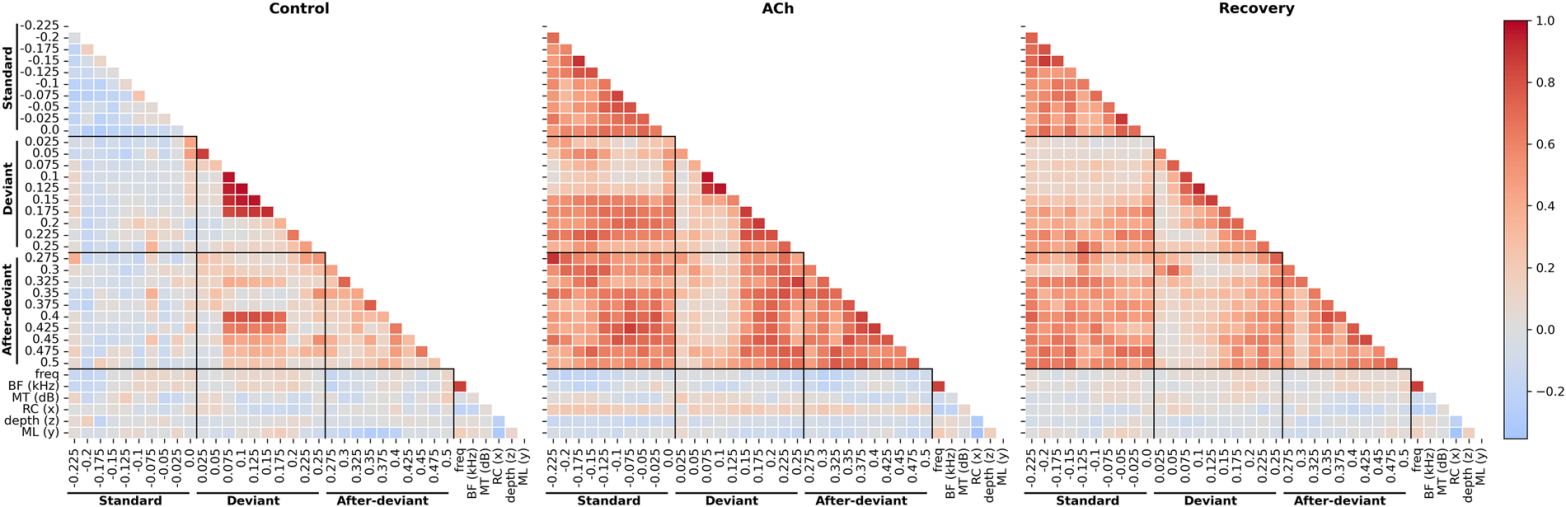
Correlation matrices showing the effects of different types of sound stimulation: FRs during standard (−0.25 to 0 seconds), deviant (0 to 0.25 s), and after-deviant (0.25 to 0.5 s) time ranges. Other columns included in the dataset were also considered in the exploratory analysis, such as stimulation frequency (freq), FRA characteristics (BF and MT) and positional information (RC, ML, depth). The mean firing rate (FR) during standard stimulation was used as the baseline for data normalization. Dark lines indicate the onset of the corresponding sound stimulation, which ends after 0.075 seconds (at −0.175 for standard, at 0.075 for deviant, and at 0.325 for after-deviant).

An exploratory analysis was also conducted using correlation matrices for the three groups: control, ACh, and recovery (Fig. 5). These correlation matrices illustrate the effects of different types of sound stimulation over time: FR during standard (from –0.250 to 0 seconds), deviant (0 to 0.250 seconds), and after-deviant (0.250 to 0.5 seconds) time ranges. Additional variables from the dataset, such as stimulation frequency (freq), FRA characteristics (BF and MF) and positional information (RC, ML, depth), were also included in the exploratory analysis. Following preliminary analyses, the mean FR during standard stimulation was selected as the baseline for data normalization, as this approach appeared to provide the most comprehensive and relevant information. However, alternative normalization criteria could reveal different patterns, offering specific insights into this phenomenon.

It is important to clarify that correlation matrices do not reflect the absolute level of neuronal activity but rather the degree of correlation between different time points, stimulation types, and other analyzed variables. Adjacent time ranges tend to show similar correlations, which is not unexpected, because the temporal dynamics of the neuronal responses are often longer than the time ranges used. In the Control matrix, a couple of clusters of high correlations are evident. One of them correspond to the range 0.075 – 0.175 seconds, which is the approximate time range of the strongest responses to the deviant stimulus. Another pattern appears in the after-deviant condition, where an increase in correlation with the stimulation with the deviant tone is observed for time points beyond 0.375. This also reflects the increase in FR in response to the after-deviant stimulus, but with longer latencies compared to the response to the deviant stimulus.

Regarding the effect of ACh on the correlation matrix, a general increase in correlation values is evident, likely due to the overall reduction in neuronal activity. Beyond this, the characteristic patterns observed in the control condition, previous to the application of Ach, are diminished. In the recovery block, a partial restoration of the correlation matrix is observed, suggesting functional recovery is starting.

## Data Availability

The datasets are provided in CSV format and are available for download on Figshare at 10.6084/m9.figshare.30132502^40^ and on GitHub at https://github.com/Vazquez-Borsetti/oddball-paradigm-in-the-auditory-cortex-an-open-dataset^41^. The raw, unprocessed data of the recordings is available at 10.12751/g-node.k9t4b5^42^, including measurements of the quality of the recordings.

## Code availability

The programs for preprocessing, data analysis, and graph generation were developed in-house using Python. The Python code was written using Python 3.9 (Python Software Foundation, https://www.python.org/). All necessary libraries (including NumPy, Pandas, Matplotlib, and Seaborn) were installed via the Anaconda distribution, and the code was primarily developed using the Spyder IDE (Integrated Development Environment). Both the database and the complete code used for the analysis are available through the link to the GitHub repository dedicated to this publication^41^.
